# Serum-circulating His-tRNA synthetase inhibits organ-targeted immune responses

**DOI:** 10.1038/s41423-019-0331-0

**Published:** 2019-12-04

**Authors:** Ryan A. Adams, Cátia Fernandes-Cerqueira, Antonella Notarnicola, Elisabeth Mertsching, Zhiwen Xu, Wing-Sze Lo, Kathleen Ogilvie, Kyle P. Chiang, Jeanette Ampudia, Sanna Rosengren, Andrea Cubitt, David J. King, John D. Mendlein, Xiang-Lei Yang, Leslie A. Nangle, Ingrid E. Lundberg, Per-Johan Jakobsson, Paul Schimmel

**Affiliations:** 1 aTyr Pharma, 3545 John Hopkins Court, Suite 250, San Diego, CA 92121 USA; 2 Division of Rheumatology, Department of Medicine, Solna, Karolinska Institutet, Karolinska University Hospital, SE-171 76 Stockholm, Sweden; 3 IAS HKUST- Scripps R&D Laboratory, Institute for Advanced Study, Hong Kong University of Science and Technology, and Pangu Biopharma, Hong Kong, China; 4 The Scripps Laboratories for tRNA Synthetase Research, 10650 North Torrey Pines Road, La Jolla, CA 92037 USA; 5 The Scripps Laboratories for tRNA Synthetase Research, Scripps Florida, 130 Scripps Way, Jupiter, FL 33458 USA

**Keywords:** Immunology, Autoimmunity, Synthetase, tRNA, HARS, Autoimmunity, Mechanisms of disease

## Abstract

His-tRNA synthetase (HARS) is targeted by autoantibodies in chronic and acute inflammatory anti-Jo-1-positive antisynthetase syndrome. The extensive activation and migration of immune cells into lung and muscle are associated with interstitial lung disease, myositis, and morbidity. It is unknown whether the sequestration of HARS is an epiphenomenon or plays a causal role in the disease. Here, we show that HARS circulates in healthy individuals, but it is largely undetectable in the serum of anti-Jo-1-positive antisynthetase syndrome patients. In cultured primary human skeletal muscle myoblasts (HSkMC), HARS is released in increasing amounts during their differentiation into myotubes. We further show that HARS regulates immune cell engagement and inhibits CD4^+^ and CD8^+^ T-cell activation. In mouse and rodent models of acute inflammatory diseases, HARS administration downregulates immune activation. In contrast, neutralization of extracellular HARS by high-titer antibody responses during tissue injury increases susceptibility to immune attack, similar to what is seen in humans with anti-Jo-1-positive disease. Collectively, these data suggest that extracellular HARS is homeostatic in normal subjects, and its sequestration contributes to the morbidity of the anti-Jo-1-positive antisynthetase syndrome.

## Introduction

Autoantibodies to aminoacyl-transfer RNA (tRNA) synthetases are key features of antisynthetase syndrome (ASS), a condition characterized by multiple organ involvement, primarily interstitial lung disease (ILD) and myositis that are often accompanied by nonerosive arthritis, Raynaud’s phenomenon, fever, and “mechanic’s hands”.^1,2^ Autoantibodies to eight different synthetases have been described, with the most common target being histidyl-tRNA synthetase, HARS; the antibodies elicited against HARS are known as anti-Jo-1 autoantibodies.^3,4^ Anti-Jo-1 autoantibodies are also the most prevalent myositis-specific autoantibody characteristic of idiopathic inflammatory myopathies (IIM, collectively termed myositis), which is a heterogeneous systemic autoimmune disease characterized by inflammation of the skeletal muscle and frequently the lungs.

IIM can be subclassified into polymyositis (PM), dermatomyositis (DM), juvenile dermatomyositis (JDM), and inclusion body myositis (IBM).^5^ ASS can also be regarded as a subtype of IIM along with the distinctly unique and recently identified subset of immune-mediated necrotizing myopathy.^6^ Anti-Jo-1-positive ASS is characterized by extensive immune cell infiltration into affected tissues.^7,8^ The pathogenesis of anti-Jo-1-positive ASS remains unknown despite a number of proposed mechanisms; there is a correlation of disease activity with antibody levels,^9^ and during disease progression, there is apparent maturation of the anti-Jo-1 response.^10,11^

An increasing number of noncanonical functions of proteins generated from aminoacyl-tRNA synthetase (aaRS) genes have been reported, demonstrating diverse roles for these proteins outside of their well-established essential function in protein synthesis. These include additional functions both inside and outside the cell that are involved in the regulation of processes such as angiogenesis, the inflammatory response, and mTOR signaling.^12–14^ A number of aaRSs, including HARS, and certain splice variants expressed from their genes have been demonstrated to be secreted from cells and have potentially important roles in the regulation of aspects of the innate and adaptive immune systems.^15–18^

Although ASS-associated glycyl-tRNA synthetase (GARS) was previously shown to be present in serum from humans and mice, other ASS autoantigens, including HARS, have not been investigated.^19^ Human HARS is a 509 amino acid homodimeric protein composed of a catalytic domain encoding residues needed for the chemistry of aminoacylation, followed by an anticodon binding domain at the C-terminal end. Like many higher eukaryote tRNA synthetases, it also harbors an appended domain that is dispensable for aminoacylation. The additional domain is designated as a WHEP domain because of the occurrence of sequence-divergent homologs in Trp-, His-, and the fused Glu-Pro tRNA synthetases. The WHEP domain is attached to the N-terminal end of human HARS, and it forms a flexible helix-turn-helix motif. Interestingly, it also appears naturally as a splice variant of HARS, encoding a domain of ~50 amino acids.^20^

Here, we demonstrate that free HARS is present in the circulation of healthy individuals, but, in contrast, it is undetectable in patients with anti-Jo-1-positive ASS. With this in mind, we were struck by the observation that circulating HARS levels are higher in anti-Jo-1-negative myositis patients, potentially because of the increased expression of HARS that is observed in regenerating muscle cells from patients with idiopathic inflammatory myopathy (IIM).^21^ This observation suggested that HARS could have a role in ameliorating inflammatory muscle conditions and led to us demonstrating that recombinant HARS was active in inhibiting T-cell activation in vitro and exhibits therapeutic activity using in vivo models of inflammatory disease.

Thus, we considered that neutralization of HARS by anti-Jo-1 antibodies would remove its protective effect against inflammatory muscle conditions. Thus, we were able to show that the generation of high-titer antibody responses to HARS in normal mice resulted in no obvious phenotype. Upon induced injury, these mice developed enhanced infiltration of immune cells into tissues, which resembled the anti-Jo-1-positive disease. Collectively, these data suggest that HARS autoantibody generation in ASS is intimately tied to activation of the immune system and perpetuates a chronic/acute inflammatory condition.

## Results

### Extracellular HARS is present in the circulation of healthy individuals and is reduced in the presence of anti-Jo-1 antibodies

Numerous splice variants and proteomic fragments are generated from the human aaRS gene family, and they have differential tissue expression and the ability to elicit a wide range of potential biological activities in cell-based assays.^22^ Previous studies showed that a number of these synthetase-derived proteins, including HARS and some of its splice variants, are secreted from cells in culture.^15^ Here, we sought to determine whether HARS was secreted in vivo and could be found in human serum. For this purpose, a sensitive immunoassay was established to quantify the levels of HARS protein. This assay used two noncompeting monoclonal antibodies that recognized the N-terminal (WHEP) domain, which is a noncatalytic appended domain specific to higher eukaryotic HARS proteins and present in all splice variants. This ECLIA (Electrochemiluminescence immunoassay) was calibrated with recombinant HARS and was sufficiently sensitive to detect single-digit pM protein levels (upper limit of quantitation: ULOQ = 2333 pM, lower limit of quantitation: LLOQ = 3.2 pM).

**Fig. 1:**
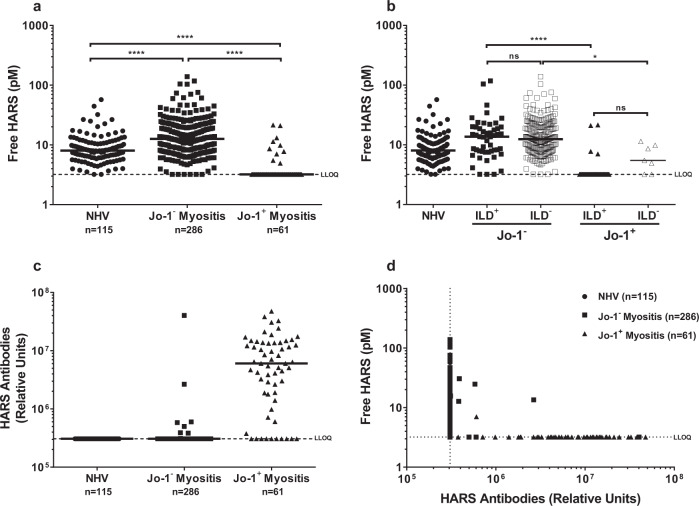
Free HARS is present in the circulation and low or absent in anti-Jo-1-positive disease. **a** Free-serum HARS levels measured in normal human volunteers (*n* = 115), anti-Jo-1-positive myositis patients (*n* = 61), and patients with anti-Jo-1-negative myositis (*n* = 286). Each dot represents an individual patient. Bars represent median values. Significantly lower levels of free HARS (****p < 0.0001) were observed in the anti-Jo-1-positive myositis patients compared to normal individuals and anti-Jo-1-negative myositis (*n* = 286) patients. **b** Serum HARS level in patients with ILD (*n* = 94) and without ILD (*n* = 243) were segmented by Jo-1 status (ILD^+^ Jo-1^−^ *n* = 50, ILD^−^ Jo-1^−^ *n* = 236, ILD^+^, Jo-1^+^ *n* = 44, ILD^−^ Jo-1^+^ *n* = 7). **c** HARS-specific antibody levels in healthy volunteers were compared to individuals with Jo-1-positive and Jo-1-negative myositis (sample numbers as in **a**). **d** Correlation of HARS serum levels with anti-HARS antibodies (data from panels **a** and **c**).

Levels of free HARS were tested in serum from a panel of both normal human volunteers (NHV, age and gender-matched) and patients with IIM with or without anti-Jo-1-positive ASS. In NHV (*n* = 115), detectable circulating free HARS was identified in 98% of the samples tested, and there was a wide range of levels (Fig. 1a). The patients with IIM were categorized according to the classification criteria most often used at the time of data collection. Some of the variables in the new classification criteria for IIM^6^ were missing in our clinical database, thus making it difficult to reclassify our patients retrospectively when applying the new criteria for IIM. In contrast, free HARS was undetectable in 84% of the serum samples tested from 61 patients with anti-Jo-1-positive IIM/ASS. The difference between the two cohorts was associated with a significance of *p* < 0.0001. In a separate analysis, free HARS in the circulation was confirmed by the dot blot technique. Although this technique is less sensitive than ECLIA, the presence of HARS was confirmed in the serum of healthy individuals, as well as in those with IIM/ASS (Supplementary Fig. 1a). Interestingly, a cohort of individuals with IMM but presenting with anti-Jo-1-negative IIM/ASS (*n* = 286) showed significant increases in their levels of circulating free HARS compared to levels of NHV (Fig. 1a). This raises the possibility that even when HARS is not an autoantigen, its circulating levels are elevated in response to muscle inflammation.

When the data were aggregated, there was no significant difference found between free HARS levels in patients with different clinical subtypes of myositis (PM, DM, IBM, and JDM). In these groups, free HARS levels correlated only with the anti-Jo-1 antibody status (Supplementary Fig. 1b). Samples were also segmented according to the presence of interstitial lung disease (ILD). For these different clinical subtypes, median levels of free HARS were determined as 6 pM for those with ILD (*n* = 94), compared to 11 pM for patients without ILD (*n* = 209); *p* < 0.0001. Of the anti-Jo-1-positive patients, 44/51 (86%) also had ILD. As a result, it is difficult to compare the free HARS levels in the anti-Jo-1-positive group simply by ILD status (Fig. 1b).

To determine whether these effects were specific for HARS, ECLIAs for asparaginyl-tRNA synthetase (NARS, the autoantigen target KS^23^) and glycyl-tRNA synthetase (GARS, the autoantigen target EJ^24^) were also used to test these samples. Both autoantigen targets were detectable in the serum of healthy controls (median = 323 pM for NARS and 120 pM for GARS) and in the patient cohorts. However, no differences in NARS or GARS levels between patient subgroups and NHV were observed for either the clinical subtype of IIM or for the anti-Jo-1-positive ASS (Supplementary Fig. 1c).

### Extracellular free HARS levels inversely correlate with anti-Jo-1 autoantibody levels

A quantitative ECLIA was also developed that was calibrated with a commercial N-terminal HARS monoclonal antibody to measure the concentration of anti-HARS autoantibody levels. This assay used coated recombinant HARS to capture circulating HARS antibodies and was followed by detection with anti-human IgG. The results were compared with those from a commercially available anti-Jo-1 autoantibody assay used to diagnose the patients.^25,26^ Seventeen percent of patients in our cohort (*n* = 357) had anti-Jo-1-positive serum (anti-Jo-1-positive myositis IIM/ASS subtypes: 100% ASS, 33% DM, 64% PM, and 1.6% JDM, Table 1). The results between the assays were largely consistent, with circulating levels of anti-HARS antibody by ECLIA detected in 51/61 anti-Jo-1-positive samples and 8/286 anti-Jo-1-negative myositis samples (Fig. 1c). Thus, anti-Jo-1 autoantibodies in serum negatively correlated with levels of free HARS (Spearman correlation *r* = −0.6244; *p* < 0.0001) (Fig. 1d). Ten of the sixty-one anti-Jo-1-positive samples had detectable levels of free HARS, nine were negative for anti-HARS antibodies by ECLIA, and one had low levels of anti-HARS antibodies. The eight anti-Jo-1-negative samples in which an anti-HARS titer was observed either had low titers of anti-HARS or HARS, which was consistent with results from the anti-Jo-1-positive population.

**Table 1:** Demographics of patient and control serum.

	IIM/ASS (*n* = 357)	Anti-Jo-1^+^ (*n* = 61)	Anti-Jo-1^−^ (*n* = 286)	NHV (*n* = 115)
Age, mean years (SD)	58 (16)	52 (15)	59 (15)^a,b^	53 (16)
Women, *n* (%)	224 (63)	40 (66)	177 (62)	69 (60)
PM/DM/IBM/JDM/IMNM, %	44/36/16/3/0.3	64/33/0/2/0	41/35/19/4/0.3	
Antisynthetase syndrome, *n* (%)	71 (20)	61 (100)	10 (4)^c^	
Interstitial lung disease, *n* (%)	94 (31)	44 (86)	50 (20)^c^	

Among the 357 IIM/ASS patients, two had unspecified myositis diagnoses, and from one patient, no specific subgroup information was available

*IIM* idiopathic inflammatory myopathies, *NHV* normal human volunteers, *PM* polymyositis, *DM* dermatomyositis, *IBM* inclusion body myositis, *JDM* juvenile dermatomyositis, *IMNM* immune-mediated necrotizing myopathy, *NA* non available, *NA ASS*–8 patients, *NA ILD* 54 patients, *NA Jo-1 status* ten patients, *within Jo-1*^*+*^ ten patients with NA ILD, *within Jo-1*^*−*^ 42 NA patients with ILD, four NA patients with ASS

^a^*p* <  0.01 vs. anti-Jo-1^+^

^b^*p* <  0.01 vs. healthy controls

^c^*p* <  0.0001 vs. anti-Jo-1^+^ (Fisher’s exact test)

### Human muscle cells secrete HARS

**Fig. 2:**
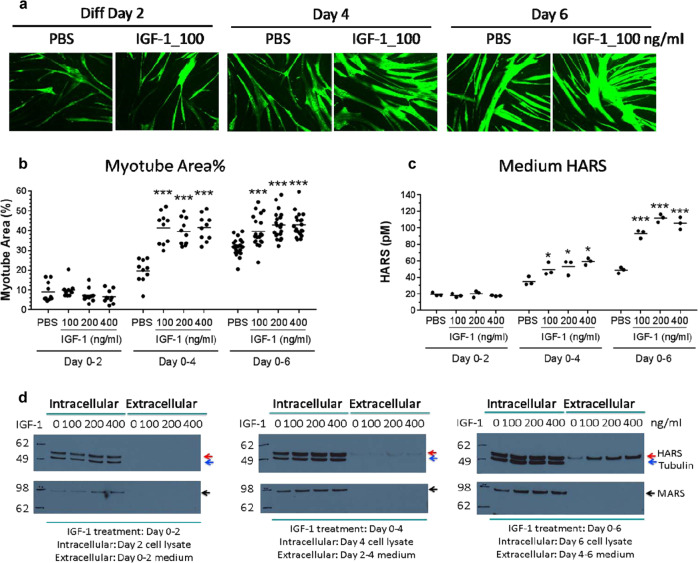
IGF-1 stimulation enhanced myotube differentiation and HARS release from HSkMC. **a** HSkMC were treated with IGF-1 or PBS during differentiation days 0–2, 0–4, or 0–6 with medium renewal every 2 days. Cells were fixed on differentiation days 2, 4, or 6 for staining (representative images shown) and analysis of myotube area (panel **b**). **c** HARS in the medium was accumulated for the last two days before harvest and was quantified by ELISA. The results are shown as the mean ± SEM. For myotube area, fusion index and nuclei number, *n* = 20 images. For HARS release, *n* = 3 biological replicates. The *p*-values of <0.05 (*) or 0.001 (***) are indicated. **d** Western blot analysis showed an increase in extracellular HARS but not MARS or Tubulin proteins in the HSkMC medium upon IGF-1 treatment.

As skeletal muscle has an overt pathology in anti-Jo-1-positive ASS patients, we tested the release of HARS from cells of muscle origin. We found that HARS was released by primary human skeletal muscle myoblasts (HSkMC) in culture and that secretion was increased during their differentiation into myotubes (Fig. 2a). Differentiation was measured by an increase in myotube area (Fig. 2b) and by the fusion index (number of nuclei within myotubes divided by total nuclei) (Supplementary Fig. 2). No significant change in the total number of nuclei was observed, suggesting that proliferation was not active during differentiation. However, HARS levels in the medium increased during differentiation (Fig. 2c, d). Western blots confirmed the release of HARS into the culture medium, and this release was specific, as there was no change in the control, i.e., methionyl-tRNA synthetase, MARS (Supplementary Fig. 2). We also observed both tubulin and MARS in cell lysates, with neither detectable in the concentrated culture medium. These data support the idea that cell lysis was not responsible for HARS release.

The release of HARS was also tested upon treatment of differentiating HSkMC with IGF-1, a known modulator of skeletal muscle growth and regeneration. As reported previously,^27^ both the myotube area and fusion index increased significantly upon the addition of IGF-1, and we found that these increases were concomitant with increases in HARS. Although IGF-1 was reported to increase muscle cell proliferation,^28^ in experiments with IGF-1, we detected no increase in the number of nuclei; moreover, we verified HARS release by western blot analysis and confirmed minimal cell lysis.

### In vivo muscle or lung injury in the presence of antibodies specific to HARS mimics features of human anti-Jo-1-positive ASS

**Fig. 3:**
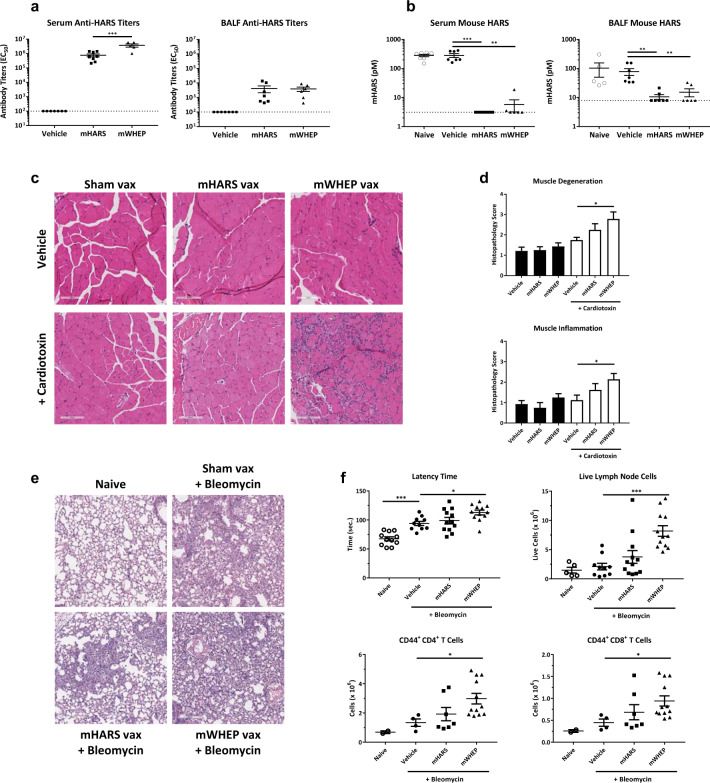
Antibodies specific to the immunomodulatory domain in mice mimic some features of human anti-Jo-1-positive antisynthetase syndrome. **a** Anti-HARS antibody titers in serum (left panel) and BALF (right panel) were measured by ECLIA. Immunized animals were all titer positive. The dotted line represents the limit of quantitation, and the bars represent the mean ± SEM. **b** Serum and BALF HARS were measured by ECLIA in control animals (naive), vehicle with control antigen, mouse HARS (mHARS), or mouse HARS WHEP domain (mWHEP). Serum HARS levels were reduced in both sets of immunizations (***p < 0.005, **p < 0.01; one-way ANOVA). BALF HARS levels were suppressed in immunized animals (**p < 0.01; one-way ANOVA). **c** Representative images of the right soleus from mice subjected to vehicle treatment (Sham Vax), mouse HARS or mouse HARS WHEP immunization followed by intramuscular (right tibialis anterior, gastrocnemius and quadriceps) injection with vehicle or cardiotoxin. Note the lack of immune cell infiltration in animals immunized with HARS WHEP in the absence of cardiotoxin, and also note the exacerbated inflammatory infiltrate in animals receiving cardiotoxin in the presence of antibodies against the HARS WHEP domain. x20 magnification. **d** Quantification of soleus muscle degeneration and inflammation based on the following semiquantitative scoring system: 0 = no significant lesion; minimal change = 1; mild change = 2; moderate change = 3; and marked change = 4. A score of 1 may, and often does, represent an incidental lesion that could be found randomly in normal animals. *p < 0.05, one-way ANOVA. **e** Representative H&E images of the lungs from animals that were naive or subjected to control immunization (Sham Vax), mHARS, or mWHEP immunization, and were then challenged with bleomycin. x10 magnification. **f** Upper left panel: the latency time required to become anesthetized by the inhaled anesthetic isoflurane was lengthened in animals challenged with bleomycin, suggesting impaired gas exchange in these animals. The latency time was even greater in animals immunized with mWHEP prior to bleomycin challenge. Upper right panel: the number of live cells isolated from the mediastinal lymph nodes of mice immunized with mWHEP and challenged with bleomycin is greater than the number present in sham-immunized bleomycin-challenged animals. Lower panels: the number of activated CD4 + (left) and CD8 + T cells (right) isolated from the mediastinal lymph nodes of mice immunized with mWHEP and challenged with bleomycin is greater than the number present in sham-immunized bleomycin-challenged animals. *p < 0.05, ***p < 0.001 one-way ANOVA followed by Dunnett’s post hoc vs. Vehicle vax/BLM challenged group.

In previous work, we demonstrated that in anti-Jo-1-positive patients, the major antibody reactivity is toward the WHEP domain of HARS.^15^ To further investigate the mechanism of autoantibody-mediated disease, we developed a model in which mice were extensively immunized to break tolerance to endogenous circulating HARS. Different strains of WT mice were found to have relatively consistent levels of circulating HARS from animal to animal, ranging from 41–149 pM in SJL/J mice (Table 2). Previous work suggested that responses are species-specific,^29^ so we disrupted the tolerance to endogenous protein by immunizing mice with murine HARS in the presence of an adjuvant. For this purpose, female SJL/J mice were immunized with full-length murine HARS or with the isolated N-terminal WHEP domain, which is a major immunodominant epitope of HARS.^15^ All animals were immunized in the presence of an adjuvant, and control mice received maltose binding protein as an unrelated immunogen (Supplementary Fig. 3a). High-titer responses were generated with anti-HARS antibodies detected in both bronchoalveolar lavage fluid (BALF) and serum of immunized animals. Interestingly, and in-line with the observation that the WHEP domain is the site of major antibody reactivity in human patient samples,^15^ in these experiments, serum titers were significantly elevated in mWHEP-immunized mice compared to the titers of mice immunized with mHARS. Both sets of titers resulted in effective neutralization of the free HARS in serum and BALF (Fig. 3a, b).

**Table 2:** HARS levels measured in serum across a panel of common laboratory mouse strains C57BL/6J & SJL/J: *n* = 20. All other strains: *n* = 10.

Strain	HARS range (pM)	HARS mean (pM)
C57BL/6J	56–429	163
SJL/J	41–149	94
CD-1	26–62	44
129SVE-M	37–368	108
A/J	69–228	115
Swiss Webster	41–133	72
TSK	84–2615	540
NSG	32–122	53

Generation of responses with either HARS or the WHEP domain alone resulted in no obvious phenotype compared to controls, and the mice demonstrated little or no additional immune cell invasion into skeletal muscle and lung. However, when immunized mice were challenged in a way that generated tissue-specific damage, by either injecting skeletal muscle with cardiotoxin or by administration of bleomycin into the lung, increased immune engagement was observed (Fig. 3c–f). Specifically, in the muscle of mice challenged with cardiotoxin, HARS WHEP-domain-specific antibodies resulted in increased muscle damage, as indicated by immune cell invasion, increased muscle fiber degeneration, and decreased muscle function (as monitored by a splay test and compared to control animals) (Fig. 3c, d and Supplementary Fig. 3b). Immune cell invasion in control animals was similar to what was seen in previous studies of cardiotoxin-induced muscle damage^30^, but was elevated in the presence of anti-HARS antibodies. Similarly, bleomycin-induced lung injury was exacerbated, and immune cell invasion into the lung increased in the absence of circulating HARS (Fig. 3e, f).

Lung function after bleomycin challenge was assessed by latency time in response to inhaled anesthetic isoflurane. The latency time was longer in mice with anti-HARS responses (Fig. 3f). Antibodies targeting HARS were also detected in the BALF of immunized animals, and free HARS was reduced compared to levels in the BALF of control animals (no antibody complex deposition was observed in lung or muscle).

In addition, 12 days after bleomycin challenge, the mediastinal lymph nodes of immunized mice had increased activated CD4 and CD8 T cells (Fig. 3f). These effects were more evident in animals immunized with the WHEP domain alone than they were in those animals immunized with the full-length protein. This finding is consistent with responses driven by the immunodominant WHEP domain of the HARS.

### HARS has immunomodulatory activity in vitro

**Fig. 4:**
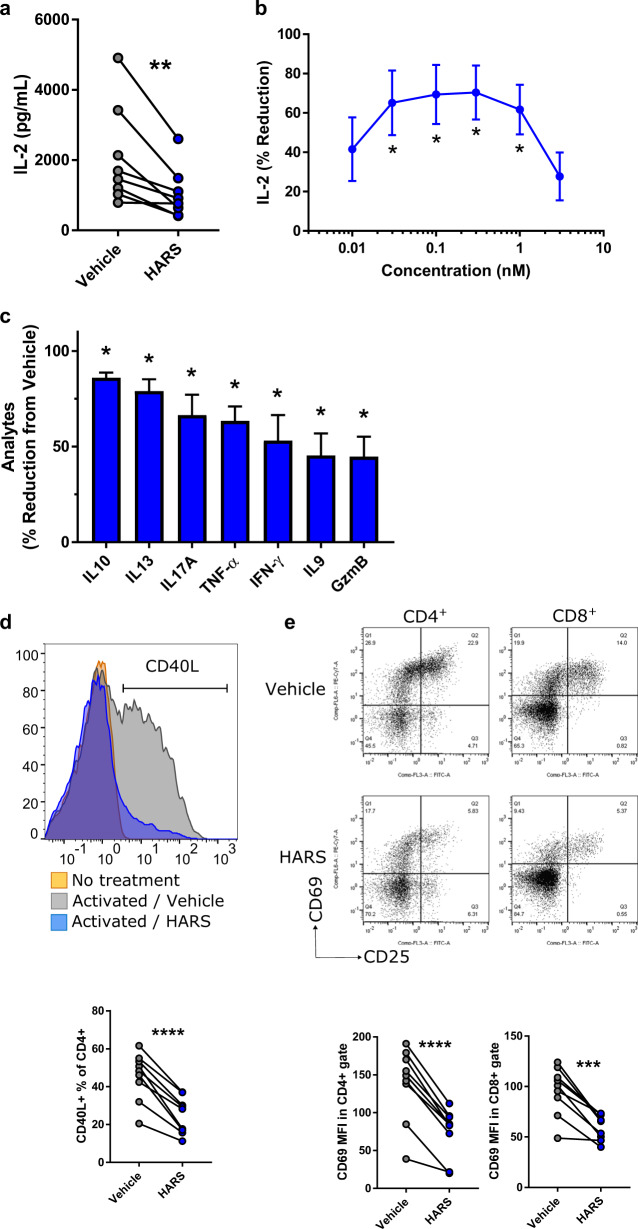
T-cell activation is reduced by HARS-related proteins. T cells isolated from PBMCs obtained from healthy volunteers were stimulated with plate-bound anti-CD3/anti-CD28 for 24 h in the presence of vehicle or recombinant human HARS.**a** IL-2 release by individual donors (*n* = 8) was measured, [HARS] = 0.3 nM. **b** IL-2 reduction by treatment with the vehicle control (*n* = 3) was measured after treatment with HARS at different concentrations. **c** Effect of HARS (0.3 nM) on the release of various cytokines and granzyme B (*n* = 2 donors in two separate experiments) were measured. **d** Flow cytometry analysis of CD40L expression on CD4 + T cells was performed, and the representative histogram and % positive results from individual donors (*n* = 9) are shown, [HARS] = 1 nM. **e** Flow cytometry analysis of CD69 expression on CD4 + (left) and CD8 + (right) T cells was performed, and representative scatter plots and MFI results from individual donors (*n* = 9) are shown, [HARS] = 1 nM. Error bars indicate SEM, paired *t*-test or ANOVA with Dunnett’s post hoc test where appropriate. *p < 0.05; **p < 0.01; ***p < 0.001, ****p < 0.0001.

Next, we investigated the in vitro effects of recombinant HARS on T cells isolated from eight independent human donors. Human recombinant HARS was expressed in *E. coli* and purified to homogeneity as near full-length proteins (a C-terminal three amino acid truncation was used to enhance solubility and production yields; endotoxin contamination was reduced to < 0.08 EU/mg). No effects of purified recombinant murine or human HARS were observed on freshly isolated resting T cells. However, HARS treatment inhibited T-cell activation induced by antibodies to CD3 and CD28 (as measured by inhibition of release of inflammatory cytokines, cell-surface markers and Granzyme B) (Fig. 4). Activation of CD4^+^ positive human T cells in the presence of human HARS resulted in inhibition of the release of interleukin (IL)-2 in 7/8 donors tested (Fig. 4a). The dose response demonstrated potent inhibition at sub-nM concentrations (Fig. 4b), which was not observed upon removal of the N-terminal WHEP domain (Supplementary Fig. 4). The resultant bell-shaped curve is consistent with a monomer-dimer equilibrium for HARS, which can form a catalytically active noncovalent dimer in solution (similar to many AARSs). Thus, monomer-dimer equilibrium is thought to regulate the functional switch from protein synthesis (dimer) to ex-translational function (monomer). A clear example of this is the work on human YARS, which has a critical role in hematopoiesis and platelet production.^31,32^ Here, the monomer-dimer equilibrium regulates ex-translational activity, with the monomer being the active signaling agent. In that work, the U- or bell-shaped curve is quite apparent and analytically consistent with a dynamic equilibrium. In this particular example, YARS self-regulates its role in stimulating hematopoiesis to avoid overstimulation at high concentrations.

As expected, the levels of cytokine release varied across T cells isolated from individual donors. However, inhibition of cytokine release by HARS was a consistent observation. Inhibition of T-cell activation was also demonstrated by reduced levels of secretion of IL-10, IL-13, IL-17A, tumor necrosis factor (TNF) alpha, interferon gamma, IL-9, and the effector molecule Granzyme B (Fig. 4c). The inhibition of activation appeared to be universal across both CD4^+^ and CD8^+^ T cells, with CD69 and CD40L upregulation inhibited in CD4- and CD8-positive T cells across all donors tested (Fig. 4d, e). In addition to classical markers of T-cell activation, HARS treatment modulated the expression of immune checkpoint receptors specifically on activated T cells (Supplementary Fig. 5).

### HARS has therapeutic activity in in vivo inflammatory disease models

**Fig. 5:**
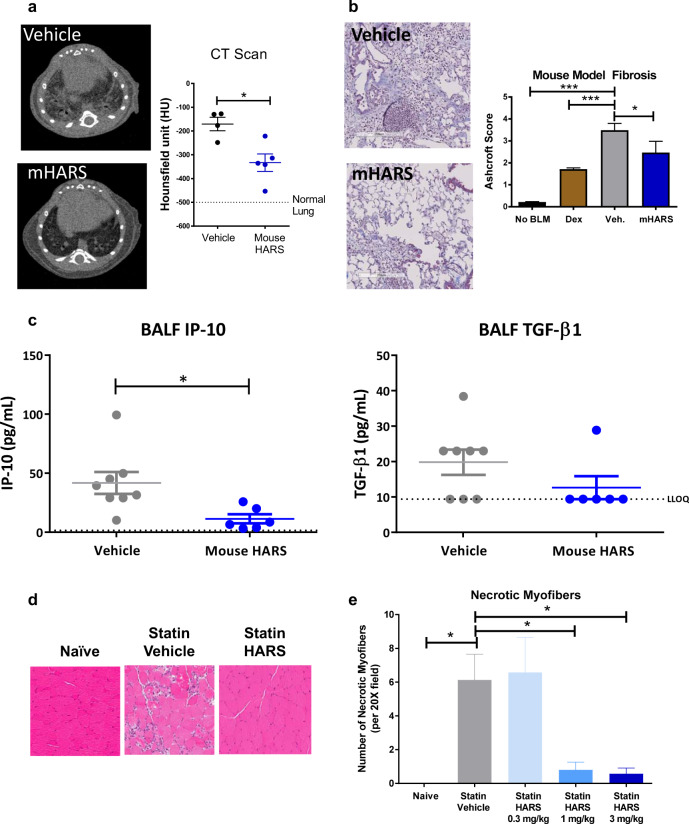
Addition of HARS can ameliorate immune-driven disease. **a** Left panel: Anatomically matched representative CT images from mice 14 days after challenge with bleomycin and treatment for 7 days with vehicle or mouse HARS. Right panel: CT scans were quantified by a mean of eight regions of interest per animal using Hounsfield units (HU). Vehicle-treated animals had an elevated mean Hounsfield unit, whereas HARS decreased the values toward those of the normal lung. **b** Representative H&E stained sections from bleomycin-induced mice terminated 21 days after challenge with bleomycin and treatment with vehicle or mouse HARS beginning on day 8. Note the immune infiltrate, fibrosis and lack of open alveoli in vehicle-treated animals and improved histology in mice treated with HARS. Right panel: Fibrosis scored on a modified Ashcroft scale in mice challenged with bleomycin on Day 0 and treated as indicated starting on Day 0 (dex) or starting on Day 8 (vehicle IV, mHARS). **c** BALF levels of IP-10 and TGFβ1 were measured from mice represented in panel **b**. **d** In a moderate statin-induced myositis model, representative histological images of hamstrings were obtained on Day 15 from animals naive to statin or receiving statin plus vehicle or statin plus HARS (3 mg/kg) beginning on Day 6 following statin initiation. **e** Necrotic fibers in statin-treated mice were counted by a person blinded to the treatment groups. HARS was administered at 0.3, 1, or 3 mg/kg on days 6–14, and muscle analysis was conducted on day 15. *p < 0.01, one-way ANOVA followed by Dunnett’s post hoc test.

Administration of recombinant HARS was tested in a number of inflammatory disease models (Fig. 5). In the well-characterized mouse intratracheal bleomycin lung injury model, recombinant murine HARS was administered 8 days after the induction of disease with bleomycin. As a positive control, the steroid dexamethasone was administered at day 0 before immune cell engagement in the model.^33,34^ HARS reduced the development of fibrosis, detectable lesions by computed tomography (CT) scan, immune cell infiltration and the presence of cytokines in the BALF (Fig. 5a–c). Significantly, the therapeutic benefit of recombinant HARS administration was opposite of the effect in mice where extracellular HARS was neutralized by high-titer anti-HARS responses, and bleomycin toxicity was exacerbated (Fig. 3).

As cerivastatin is known to cause skeletal muscle damage at high doses in both rodents and people,^35–37^ HARS was tested for its therapeutic activity in a skeletal muscle rodent model where high-dose treatment with cerivastatin induces skeletal muscle damage and immune cell invasion. Skeletal muscle, such as lung, is significantly compromised in anti-Jo-1-positive antisynthetase patients and results in skeletal muscle inflammation and myositis. We induced severe myositis with daily administration of cerivastatin to adult male rats. All animals challenged with statin that were receiving a vehicle required euthanasia by Day 14. In contrast, intervention with HARS beginning on Day 6 decreased morbidity and improved survival in a dose-related manner (Supplementary Fig. 6a). Less aggressive dosing of cerivastatin (daily dosing on Days 0–7, every other day on Days 7–15) caused significant skeletal muscle tissue damage but no lethality for >15 days. HARS administered starting at day 7 reduced tissue damage, immune cell infiltration, and the expression of genes related to inflammation in a dose-responsive fashion (Fig. 5d, e and Supplementary Fig. 6b, c). Collectively, these results in mice and rats support further investigation of HARS and its WHEP domain for therapeutic applications.

## Discussion

Since 1983, anti-Jo-1-positive ASS patients have been known to have circulating antibodies to the intracellular protein HARS, which is the aminoacyl-tRNA synthetase specific for histidine. A number of potential mechanisms have been suggested to explain the role of these antibodies in the pathology of disease based on the association between clinical features and antibody titers.^9–11^ Here, we demonstrate that extracellular HARS is in circulation in normal individuals and is absent or reduced in anti-Jo-1-positive ASS patients. Free HARS levels in the circulation were reduced to undetectable levels in ~84% of anti-Jo-1-positive ASS individuals, and the levels were inversely correlated with levels of anti-Jo-1 (anti-HARS) antibodies. This finding prompts a number of questions, including whether circulating HARS has a functional role in healthy individuals and whether its neutralization in ASS is relevant to disease pathology.

In early work, Howard et al.^38^ reported that HARS and NARS activate chemokine receptors on T lymphocytes and immature dendritic cells. Given that more than 15 years have passed since these studies were published and the lack of subsequent investigations, we attempted to repeat the work with HARS. In spite of several attempts, we could not verify the results. For this reason, we moved on to study the activities of HARS independently of any bias toward earlier work. While our studies reached a different conclusion, we cannot rule out that the mechanisms reported by Howard et al.^38^ act in addition to what we studied here.

HARS was demonstrated to inhibit T-cell activation in vitro, and the circulating levels of this protein detected in healthy individuals suggest that naturally circulating levels of HARS might be sufficient to contribute to the regulation of immune cell activation in multiple tissues. In addition, HARS is released from muscle cells in a regulated fashion, suggesting that HARS release might enable an immune regulatory mechanism. Immune cells are chronically activated in anti-Jo-1-positive ASS, and T cells are known to infiltrate certain tissues, especially skeletal muscle and lungs. Anti-Jo-1-positive ASS patients have no or low circulating levels of free HARS, consistent with the loss of a HARS-based regulatory mechanism.

Two recent reviews explored the role of T cells in models of muscle disease. These reviews highlight the involvement of T cells in conditions of myopathy.^39,40^ In addition, recent work has shown that CD8 T cells actively infiltrate muscle upon cardiotoxin treatment.^41^ Through a mechanism of regulating MCP-1 secretion, these same CD8 T cells impact the further recruitment of macrophages. In our work, we explored both cytokine release and gene expression changes in human macrophages in response to HARS treatment in vitro. We observed minimal effects. On the other hand, the immunomodulatory activity of HARS directly impacts activated T cells, which in turn may directly or indirectly affect the infiltration and activity of macrophages in muscle. Downstream effects of impaired immune cell infiltration, such as muscle regeneration and matrix deposition, are important areas for future studies. While we acknowledge that the system is complex, our experiments aimed at deconvoluting the system and more clearly defining the role of HARS, and our results support the idea that T-cell infiltration is a significant causal factor in HARS production.

Although our data support the conclusion that the antibodies are induced by a T-cell-dependent mechanism, the reason that tolerance is disrupted in some people and not others remains elusive. At this point, we cannot explain the ability of HARS immunization (in the presence of adjuvant) to induce (presumably) T-cell-dependent anti-HARS antibodies. An explanation not explored is that more purified forms of HARS could induce antigen-specific regulatory T cells (in the absence of adjuvant) that may manifest bystander suppression. This possibility is an important consideration, but at this point, we have exhausted our ability to go beyond the high levels of purity of HARS that we already have in hand. Furthermore, we performed in vitro T regulatory induction and suppression assays with HARS. The results were inconclusive, and we did not further pursue this issue. Last, in the data in Fig. 3, the deleterious effects of HARS sequestration in the muscle and lung injury models are clear. In a simple scenario, HARS sequestration would be necessary and sufficient for the observed exacerbation of the induced injury. Additionally, or alternatively, the induced injury could have enhanced exposure of HARS in muscle or lung resulting in HARS-specific T cells infiltrating the target tissues to induce inflammation.

Previous attempts to reproduce features of anti-Jo-1-positive antisynthetase syndrome through immunization of mice with HARS have resulted in mixed findings.^29,30^ Immunization with HARS protein resulted in no obvious lymphocyte infiltration into muscle or lung tissue, yet direct intramuscular injection of an expression plasmid for HARS did provoke experimental myositis in the injected tissue.^42^ Similarly, our data revealed that high-titer responses to HARS in mice have little effect on pathology, yet upon tissue challenge, there is notably increased immune cell infiltration. This effect is marked in mice with high-titer responses to HARS, especially to the WHEP domain of the protein. Conversely, administration of recombinant HARS has a therapeutic effect in disease models where relevant tissues have an overt anti-Jo-1-driven pathology, specifically muscle and lung. Both the bleomycin-induced lung injury and the statin-induced myopathy models are driven by initial inflammation with a significant T-cell component. The therapeutic activity of HARS is likely a result of an immune-related function rather than the canonical role of HARS in protein synthesis. Secretion of HARS proteins by muscle cells further suggests a potential role in maintaining tissue-specific homeostasis by modulating the immune system at the site of damage.

A few reports support a possible role exerted by anti-Jo-1 antibodies in the pathogenesis of IIM/ASS; however, no functional studies have been published thus far.

In a separate vein, we recently showed that anti-Jo-1 antibodies display an IgG Fc-glycan profile normally associated with anti-inflammatory properties.^43^ Complement 5b-9 deposits were observed in capillaries located near the perimysium area in muscle biopsies of Jo-1+ patients. This may be an indication of immune activation with antibody and T-cell involvement.^44^ Thus, we cannot exclude a possible independent pathogenic role for anti-Jo-1 antibodies in IIM/ASS, such as small, pro-inflammatory immune complexes composed of HARS and anti-Jo-1 antibodies.^10,45^ However, in our work in mouse and rodent systems, the administration of HARS showed a clear therapeutic benefit. This suggests that regardless of an independent role of the antibodies in the disease etiology, the sequestration of HARS is a major contributor.

Our data show that, at least in the mouse, HARS had immune-suppressive effects in the bleomycin and statin models (Fig. 5 and S6). This observation suggests that therapeutic benefits could come from the use of HARS in other subsets of inflammatory myositis. In Fig. 1, we show that serum levels of HARS in NHV span a large range of ~3 to 60 pM, with a median value of ~8 pM. In a larger set of Jo-1- myositis patients, the median value was raised to ~17 pM (twofold higher), with a few outliers (out of 286 subjects) reaching just over 100 pM. Thus, if HARS is active in principle as an immunosuppressant for Jo-1- myositis, then a 9 pM increase in levels may be insufficient for a therapeutic benefit. Additionally, in the case of myositis patients who have a Jo-1- ILD + comorbidity, serum HARS levels trend higher than they do in NHV but are not significantly different than those from Jo-1- ILD- patients. This suggests a lack of an internal regulatory mechanism to increase HARS levels in the setting of Jo-1- ILD + disease.

A number of aaRSs in addition to HARS are the targets of autoantibodies in different presentations of ASS. Although anti-HARS has by far the highest frequency in clinical presentations, associations of ASS with autoantibodies directed against AlaRS (PL-12), ThrRS (PL-7), IleRS (OJ), AsnRS (KS), GlyRS (EJ), TyrRS (Ha), and PheRS have been reported (ZO).^1^ Several have been implicated in novel functions in inflammatory responses.^46^ We chose the HARS system because it is the most prominent, but our work here motivates a deeper analysis of the other aaRSs. If some or all of these presentations come from circulating tRNA synthetase-sequestration, then in a clinical setting, therapeutic interventions can be tailored to a specific synthetase. While HARS might not be causally involved in all subgroups of myositis, our data in mouse and rodent models (Fig. 5) suggest that inflammatory conditions not caused by ASS could potentially be treated by HARS administration. By extension, HARS might also be of benefit for cases where a different synthetase is involved in the disease mechanism.

## Methods

### Patient samples

Serum samples from 357 patients with IIM/ASS were collected at the Rheumatology clinic at Karolinska University Hospital, Stockholm, Sweden.^47,48^ By measuring the presence of anti-Jo-1 antibodies at least once during the disease course, 61 anti-Jo-1-positive and 286 anti-Jo-1-negative patients were identified. Out of 357 patients, 71 patients (20%) fulfilled the criteria for ASS, and 94 patients (31%) presented with ILD. Serum samples from 115 age- and gender-matched normal human volunteers were also included. Major demographic data are summarized in Table 1.

### Preparation of recombinant HARS

Recombinant HARS (amino acids 1–506) was expressed as a soluble protein in *E. coli* without tags. HARS (1–506) was purified from lysed *E. coli* cells by anion exchange chromatography (Q-Sepharose HP) at pH 7.4 with elution by a gradient of increasing NaCl concentration, followed by hydrophobic interaction chromatography with phenyl Sepharose HP at pH 7.0 using a reverse gradient of ammonium sulfate. The protein was further purified using ceramic hydroxyapatite chromatography at pH 7.0 and was eluted by an increasing sodium phosphate concentration, after which the protein was buffer exchanged into 20 mM sodium phosphate, 150 mM NaCl pH 7.0, and sterile filtered for storage at −80 °C. Purity was analyzed by sodium dodecyl sulfate–polyacrylamide gel electrophoresis and size-exclusion chromatography, which demonstrated > 95% purity and less than 3% high-molecular weight material. The endotoxin level was determined using an LAL assay (Charles River) and shown to be less than 1 EU/mg.

### Measurement of human endogenous HARS

An ECLIA detection assay was developed to quantitate the levels of human HARS in plasma or serum using a Meso Scale Diagnostics (MSD) platform. Quantitation was achieved using capture and detection antibodies targeting the N-terminal domain of HARS (approximately amino acids 1–60 of HARS), and it was calibrated with highly purified recombinant HARS. Assays were conducted using a 96-well multi-array plate coated with a mouse monoclonal capture antibody (clone 12H6) following standard Meso Scale Diagnostics protocols and using a biotinylated mouse monoclonal antibody (clone 1C8) for detection.

An ELISA was developed to detect and quantitate the levels of human HARS in muscle cell supernatants. The assay utilized anti-HARS monoclonal antibodies M03 and biotinylated-M01 in a sandwich to measure HARS concentrations in all the sample media collected in this study.

For dot-blots, serum (1 μL) was applied to nitrocellulose membranes, and after drying, the membranes were blocked with 5% nonfat milk in phosphate-buffered saline (PBS) containing 0.1% Tween. Blots were probed with monoclonal mouse anti-human HARS N-terminal (Abcam) and developed with a polyclonal goat anti-mouse IgG HRP conjugate (Dako) followed by enhanced chemiluminescent detection.

### Measurement of HARS-specific (Jo-1) antibodies

An ECLIA to detect HARS-specific antibodies was developed by coating recombinant HARS and incubating diluted serum (1:1,000,000) on the HARS capture plate. HARS-reactive antibodies were detected with Sulfo-tagged goat anti-human IgG (MSD Cat. #R32AJ-1). Antibody levels were compared to standard curves of a mouse monoclonal antibody (clone 1C8) and were described in relative units.

### Release of HARS from human muscle cells

Adult human skeletal muscle cells (HSkMC, Cell Applications) were seeded at 40,000/cm^2^ on collagen-coated plates and grown in growth medium for 24 h before changing to differentiation medium. The first day in differentiation medium was regarded as Diff Day 0. Medium was renewed every 2 days, and media was harvested as 0–2, 2–4, and 4–6 samples, with three biological replicates per condition. Cells were fixed on differentiation days 2, 4, or 6 and stained for myotubes using anti-human myosin antibody; nuclei were stained with Hoechst 33342 nuclear stain. The myotube area was analyzed by determining the fusion index (calculated as the number of nuclei within the myotube-stained area over the total number of nuclei) and the nuclear number per imaging field, with a total of 20 images from two biological replicates. HARS ELISA was used to quantify HARS in the medium samples. HARS, MARS and Tubulin proteins in whole-cell lysates (intracellular) and in concentrated medium (extracellular) were analyzed by western blotting using specific antibodies.

### T-cell activation

Human whole blood was collected from healthy donors and processed within 2 h of collection. Blood was diluted 1:1 in PBS and was layered onto Lymphoprep (StemCell Technologies) in Leucosep centrifuge tubes (Greiner Bio-One), and peripheral blood mononuclear cells (PBMC) were isolated by density-gradient centrifugation. PBMCs were washed twice in PBS with 2% fetal bovine serum (FBS, ATCC), and T cells were purified using a negative selection magnetic kit (StemCell Technologies) following the manufacturer’s instructions. The purity of the T cells was > 95%.

Ninety-six-well flat-bottomed plates were coated with 1.25 to 5 μg/mL of anti-CD3 antibodies (BioLegend, clone UCHT-1) overnight or for 2 h at 37 °C. After 2 washes with PBS, T cells (100,000 cells/well) were added along with soluble anti-CD28 antibody (BD Biosciences, clone CD28.2) at 1 μg/mL final concentration; tests were performed in complete RPMI medium (RPMI 1640 (ATCC), 10% FBS, 50 μg/mL gentamycin (Thermo Fisher Scientific), 1/100 MEM nonessential amino acids (Thermo Fisher Scientific)). Each condition was assessed in triplicate. After 24 h of incubation at 37 °C, the supernatants were collected and immediately analyzed or stored at −80 °C until analysis by ELISA. IL-2 was measured by an MSD kit (Meso Scale Diagnostics), and IL-10, IL-13, IL-17A, TNF-α, IFNγ, and IL-9 were analyzed with a multiplex cytokine assay (EMD Luminex). ELISAs (Thermo Fisher Scientific) were used to quantify Granzyme B levels. For flow cytometry, T cells were incubated on ice with an FcR Blocking Reagent (Miltenyi Biotec) for 10 min and then stained with anti-CD4 BV510, anti-CD8 APC/Cy7, anti-CD69-PE/Cy7, and anti-CD40L-BV421 (all from BioLegend). Propidium iodide (Miltenyi Biotec) was added as a viability marker. Samples were acquired on a MACSquant flow cytometer (Miltenyi Biotec) and analyzed with FlowJo software (Tree Star).

T-cell checkpoint receptor expression experiments were performed at Primity Bio (Fremont, CA) and utilized their surface profiling platform. Primary human PBMCs were stimulated with α-CD3/CD28 (Biolegend; 1 µg/mL) and treated with HARS (0.1 nM) for 24 h. Data are represented as the percentage of positive T cells.

### Animal studies

Studies conducted in animals were performed following prior approval of local institutional animal care and use committees (IACUCs) and were in accordance with accepted standards to ensure animal welfare. Housing areas were controlled for temperature and light cycle (12L:12D), with water and standard laboratory chow available ad libitum.

### Immunization studies

Female SJL/J mice (The Jackson Laboratory, 8 weeks of age) received 200 µg of antigen in a 100 µL vehicle volume (20 mM NaPO4, 0.15 M NaCl, pH 7.0) or vehicle alone (Sham Vax); the vehicle was emulsified with 100 µL complete Freund’s adjuvant (CFA, Difco) and was introduced subcutaneously at two different sites (e.g., flank, base of tail; 100 µL per site). Subsequent immunizations at 2-week intervals were conducted with 200 µg of antigen in 100 µL vehicle emulsified with 100 µL incomplete Freund’s adjuvant (IFA, Difco). In independent experiments, 7 weeks after the initial immunization, animals were challenged by oropharyngeal administration of bleomycin (2 U/kg, Hospira, Lot: B051485AA) or intramuscular administration of cardiotoxin (10 µg per injection; 50 µL of 0.2 mg/mL solution) in the quadriceps, gastrocnemius and tibialis anterior muscles of one side (Sigma Cat. #C9759*)*. Corresponding controls received vehicle treatments in the same manner.

Lung function was assessed 1 week after challenge by placing mice individually in a chamber containing 2% isoflurane and monitoring the latency of anesthetic. An increased latency time is hypothesized to result when gas exchange at the alveolar surface is impaired. Muscle function was assessed 6 days after challenge by evaluating the degree to which mice splayed the rear legs when picked up by the tail. In healthy animals, rear legs were extended far from the body (score = 0), whereas in animals with impaired muscle function, the foot remained close to the body (score = 2).

At termination (12 days after lung challenge or 7 days after muscle challenge), serum and BALF were harvested for measuring HARS and antibody titers; tissues were collected, fixed, processed to produce slides, stained with H&E and scored by a pathologist. Mediastinal lymph nodes were collected for immunophenotyping.

### Bleomycin-induced lung injury

On day 0, female C57bl/J mice were anesthetized with pentobarbital sodium (Kyoritsu Seiyaku, Japan) and intratracheally administered bleomycin (Nippon Kayaku, Japan) in PBS saline at a dose of 3 mg/kg in a volume of 50 µL per animal using a Microsprayer^®^ (Penn-Century, USA). Mouse HARS was intravenously administered at a dose of 3 mg/kg once daily from day 8 to 21. Dexamethasone was orally administered to the mice at doses of 0.25 mg/kg once daily from day 0 to 21. CT scans were performed on 4 or 5 randomly selected mice from each group. The mice were mounted on a holder and placed in an X-ray CT system (LCT-200, Aloka, Japan) under pentobarbital sodium anesthesia. Images were converted into a DICOM format and analyzed with Onis Viewer (DigitalCore, Japan). Two sections (upper: forth dorsal vertebra, lower: seventh dorsal vertebra) were analyzed from each scan data set, and eight regions of interest (ROI) were defined in the following areas: the right upper anterior and posterior regions and the left upper anterior and posterior region. The means of the intensity of the eight ROIs were defined as an individual’s level of lung density. Termination was conducted on day 21. BALF was collected and centrifuged, and the supernatant was collected and stored until biomarker analysis. Lungs were harvested, processed to produce slides and stained with Masson’s trichrome. The degree of pulmonary fibrosis was evaluated using the Ashcroft score^49^ for the quantitative histological analysis.

### Statin model

Ten-week-old female Sprague-Dawley rats (*n* = 8 per group) were dosed daily with 1 mg/kg cerivastatin for 7 days and then every other day (qod). Cerivastatin was diluted in 0.5% methylcellulose and administered by oral gavage. On days 6–14, HARS was administered intravenously at doses of 0.3, 1, or 3 mg/kg. In parallel, negative controls received the vehicle (20 mM NaPO4, 0.15 M NaCl, pH 7.0). All rats were euthanized on day 15, and hamstring muscles were harvested, fixed, processed to produce slides, and stained with H&E. A pathologist (Premier Laboratory) blinded to the treatment groups assessed myofiber degeneration/necrosis. Necrosis was characterized by swollen or condensed fibers with or without fragmentation, and often with altered staining (usually increased eosinophilia). A subjective, semiquantitative scoring system was utilized by the Pathologist: 0 = no significant lesion; minimal change = 1; mild change = 2; moderate change = 3; and marked change = 4. Subsequently, the sections were quantified for necrotic myofibers and the number of nuclei per 20x field using imaging software at aTyr Pharma by a scientist blind to treatments. The number of nuclei was measured as an indication of inflammatory cells infiltrating the muscle.

### Cardiotoxin-induced myositis

Cardiotoxin extracted from *Naja mossambica mossambica* was injected into the tibialis anterior, quadriceps and gastrocnemius muscles of one mouse hind limb (Sigma Cat. #C9759; 10 µg/injection). The contralateral limb was injected with saline and served as a control. Tissues were harvested 7 days after cardiotoxin administration and were then stained with hematoxylin and eosin.

### Transcriptional profiling of rat hamstring tissue by quantitative PCR (qPCR)

Rat hamstring tissue was excised from animals, frozen in liquid nitrogen and stored at −80 °C until analysis. Tissue was prepared using Qiagen’s RNeasy Fibrous Tissue Midi Kit (Catalog #75742). Once RNA was eluted from the Qiagen column, it was run on an Agilent’s Bioanalyzer 2100 to test RNA integrity and on a NanoDrop to determine RNA concentration and purity. RNA was then stored at −80 °C.

Reverse transcription (RT) of RNA to complementary DNA (cDNA) was performed in a 96-well PCR plate in an Eppendorf’s Mastercycler PCR machine with the following program: 37 °C for 60 min and 95 °C for 5 min. The edge wells of the 96-well plate were not used and were filled with 50 µL of water to prevent evaporation of the inside wells. A total of 20 µL of RNA and 30 µL of reverse transcription master mix (Ambion’s TaqMan Pre-Amp Cells to CT Kit catalog #4387299) were used per sample RT. Once RT was completed, the next step was to preamplify genes of interest in the sample cDNA. Primers of genes of interest (DELTAgene primers designed by Fluidigm; Table 3) were combined to a final concentration of 200 nM. Using these primers, genes of interest were preamplified in each sample. Preamplification was performed in 10 µL reactions (2.5 µL cDNA, 7.5 µL Pre-Amp mastermix) in a 384-well format using Applied Biosystem’s ViiA7 PCR machine with the following program: 95 °C for 10 min, 14 cycles of 95 °C for 15 seconds and 60 °C for 4 min. After the preamplification step, exonuclease (New England BioLabs catalog #M0293L) was added to remove unincorporated primers from each sample. This exonuclease reaction was also completed in the ViiA7 PCR machine with the following program: 37 °C for 30 min and 80 °C for 15 min. After the exonuclease reaction, the RT sample was further diluted 1:5 (7 µL exonuclease sample + 18 µL low-EDTA buffer).

**Table 3:** Gene panel for muscle qPCR.

Gene panel	Genes
Muscle-associated	*Acta1, Actb, Acvr2b, Adrb2, Agrn, Akt1, Akt2, Atp2a1, B2m,Bcl2, Bhlhe41, Bmp4, Camk2g, Capn2, Capn3, Casp3, Cast, Cav1, Cav3, CD138, CD8b, Cryab, Cs, CTGF, Ctla4, Ctnnb1, CYCS, Dag1, Dars, Des, DLEC, Dmd, Dysf, Emd, Fbxo32, FOXO1, Foxo3, FSP1, Gapdh, GARP, Gars, GITR, Gpr44, Gusb,, Hars, Hars2, Hk2, Hprt1, Hsp90ab1, Igf1, Igf2, Igfbp3, Igfbp5, Ikbkb, Il17ra, Il17rb, Il17rc, Il17re, Ldha, Lep, Lmna, LTA, Mapk1, Mb, Mbnl1, Mef2c, Mstn, Musk, Myf5, Myf6, Myh1, Myh2, Myod1, Myog, Myot, Neb, Nos2, Pax3, PAX6, PAX7, Pdk4, Ppp3ca, PRF1, Ptprc, Qars, Rhoa, Rora, Rplp1, Rps6kb1, Runx3, Sgca, TRIM63, Utrn*
Immune-associated	*B2m, Ccl20, Ccl5, Ccr4, CCR5,Ccr6, CD117, CD11A, CD11b, CD127, CD14, CD18, CD19, CD20, CD25, CD28, CD29, CD3, CD4, CD45R, CD49D, CD8a, CD8b1, COL1A2, COL3A1, CXCR3, EBI3, F4/80, Fasl, FGF1, Fgf2, Foxp3, FZD7, Gapdh, Gata3, Gata4, Hars, Hsp90ab1, ICAM1, ICOS, Ifgr, IFNa1, IFNg, IKZF2, IL10, IL12A, IL12B, IL12RB1, IL12RB2, IL13, IL15, IL15RA, IL17a, Il17c, Il17d, Il17f, IL18RA, Il1b, IL1r, Il2, Il21, Il22, Il23a, Il23r, Il25, Il27, Il2ra, IL33r, Il4, Il4ra, IL5, Il6, Il6ra, Itgax, MAF, MCP1, MIF, MMP1, MMP3, Mmp9, NCAM, Nfkb1, NK1.1, Rig1, RORC, Stat1, Stat3, Stat4, Stat5a, Stat6, TBX21, Tgfb1, Tgfb3, Tnf, VCAM1, WNT7a*

The chip used to run qPCR on Fluidigm’s Biomark system was a 96.96 Dynamic Array IFC for Gene Expression. The chip was first primed with the IFC controller HX according to the manufacturer’s recommendations before sample and assays were loaded. To prepare assays to be loaded on a chip, 4.4 µL of assay master mix (Fluidigm’s 2x Assay Loading Reagent catalog #8500736 and low-EDTA TE) and 3.6 µL of 20 µM forward and reverse primers for each gene of interest were prepared in a 96-well plate. To prepare samples, 4.5 µL of sample master mix (Ambion’s 2x TaqMan Gene Expression Master Mix, Fluidigm’s 20x DNA Binding Dye Sample Loading Reagent catalog number 100-0388, and Biotium’s 20x EvaGreen catalog #31000) was added to 3 µL of diluted preamplified/exonuclease sample in a 96-well plate. Once the chip had been primed, 5 µL of each sample was loaded onto the chip. The chip was then returned to the IFC controller for the samples to be loaded into the chip. After the chip had finished loading, qPCR could then be run on a Biomark system using a preset program for 96.96 Dynamic Array for Gene Expression with a melt curve to determine primer specificity. Relative gene expression was determined by the delta Ct (ΔΔCt) method.

## Supplementary information

Supplementary Legends

Supplementary Figure 1

Supplementary Figure 2

Supplementary Figure 3

Supplementary Figure 4

Supplementary Figure 5

Supplementary Figure 6

## References

[1] Mahler M, Miller FW, Fritzler MJ (2014). Idiopathic inflammatory myopathies and the anti-synthetase syndrome: a comprehensive review. Autoimmmun. Rev..

[2] Hervier B, Benveniste O (2013). Clinical heterogeneity and outcomes of antisynthetase syndrome. Curr. Rheumatol. Rep..

[3] Mathews MB, Bernstein RM (1983). Myositis autoantibody inhibits histidyl-tRNA synthetase: a model for autoimmunity. Nature.

[4] Ramsden DA (1989). Epitope mapping of the cloned human autoantigen, histidyl-tRNA synthetase. Analysis of the myositis-associated anti-Jo-1 autoimmune response. J. Immunol..

[5] Amato AA, Greenberg SA (2013). Inflammatory myopathies. CONTINUUM: Lifelong Learn. Neurol..

[6] Lundberg IE, de Vissier M, Werth VP (2018). Classification of myositis. Nat. Rev. Rheumatol..

[7] Miller FW, Love LA, Barbieri SA, Balow JE, Plotz PH (1990). Lymphocyte activation markers in idiopathic myositis: changes with disease activity and differences among clinical and autoantibody subgroups. Clin. Exp. Immunol..

[8] Hervier B (2016). Involvement of NK cells and NKp30 pathway in antisynthetase syndrome. J. Immunol..

[9] Stone KB (2007). Anti-Jo-1 antibody levels correlate with disease activity in idiopathic inflammatory myopathy. Arthrit. Rheuma..

[10] Miller FW, Twitty SA, Biswas T, Plotz PH (1990). Origin and regulation of a disease-specific autoantibody response. Antigenic epitopes, spectrotype stability, and isotype restriction of anti-Jo-1 autoantibodies. J. Clin. Invest..

[11] Ascherman DP (2015). Role of Jo-1 in the immunopathogenesis of the anti-synthetase syndrome. Curr. Rheumatol. Rep..

[12] Xu X (2012). Unique domain appended to vertebrate tRNA synthetase is essential for vascular development. Nat. Commun..

[13] Park SG, Schimmel P, Kim S (2008). Aminoacyl tRNA synthetases and their connections to disease. Proc. Natl Acad. Sci. (USA).

[14] Arif A (2017). EPRS is a critical mTORC1-S6K1 effector that influences adiposity in mice. Nature.

[15] Zhou JJ (2014). Secreted histidyl-tRNA synthetase splice variants elaborate major epitopes for autoantibodies in inflammatory myositis. J. Biol. Chem..

[16] Kron MA, Metwali A, Vodanovic-Jankovic S, Elliott D (2013). Nematode asparaginyl-tRNA synthetase resolves intestinal inflammation in mice with T cell transfer colitis. Clin. Vacc. Immunol..

[17] Ahn YH (2016). Secreted tryptophanyl-tRNA synthetase as a primary defence system against infection. Nat. Microbiol..

[18] Kim SB (2017). Caspase-8 controls the secretion of inflammatory lysyl-tRNA synthetase in exosomes from cancer cells. J. Cell Biol..

[19] Park MC (2012). Secreted human glycyl-tRNA synthetase implicated in defense against ERK-activated tumorigenesis. Proc. Natl Acad. Sci. (USA).

[20] Xu Z (2012). Internally deleted human tRNA synthetase suggests evolutionary pressure for repurposing. Structure.

[21] Casciola-Rosen L (2005). Enhanced autoantigen expression in regenerating muscle cells in idiopathic inflammatory myopathy. J. Exp. Med..

[22] Lo WS (2014). Human tRNA synthetase catalytic nulls with diverse functions. Science.

[23] Hirakata M (1999). Anti-KS: identification of autoantibodies to asparaginyl-transfer RNA synthetase associated with interstitial lung disease. J. Immunol..

[24] Targoff IN (1990). Autoantibodies to aminoacyl-transfer RNA synthetases for isoleucine and glycine. Two additional synthetases are antigenic in myositis. J. Immunol..

[25] Targoff IN, Reichlin M (1987). Measurement of antibody to Jo-1 by ELISA and comparison to enzyme inhibitory activity. J. Immunol..

[26] Nishikai M, Reichlin M (1980). Heterogeneity of precipitating antibodies in ploymyositis and dermatomyositis. Characterization of the Jo-1 antibody system. Arthrit. Rheuma..

[27] Rommel C (2001). Mediation of IGF-1-induced skeletal myotube hypertrophy by PI(3)K/Akt/mTOR and PI(3)K/Akt/GSK3 pathways. Nat. Cell Biol..

[28] Florini JR, Ewton DZ, Coolican SA (1996). Growth hormone and the insulin-like growth factor system in myogenesis. Endocr. Rev..

[29] Katsumatu Y (2007). Species-specific immune responses generated by histidyl-tRNA synthetase immunization are associated with muscle and lung inflammation. J. Autoimmun..

[30] Sciorati C (2014). 7-Tesla magnetic resonance imaging precisely and non-invasively reflects inflammation and remodeling of the skeletal muscle in a mouse model of anti-synthetase syndrome. Biomed. Res. Int..

[31] Kanaji T (2018). Tyrosyl-tRNA synthetase stimulates thrombopoietin-independent hematopoiesis accelerating recovery from thrombocytopenia. Proc. Natl Acad. Sci. USA.

[32] Vo MN, Yang XL, Schimmel P (2011). Dissociating quaternary structure regulates cell-signaling functions of a secreted human tRNA synthetase. J. Biol. Chem..

[33] Wynn TA (2011). Integrating mechanisms off pulmonary fibrosis. J. Exp. Med..

[34] Jenkins RG (2017). ATS assembly on respiratory cell and molecular biology. An Official American Thoracic Society Workshop Report: use of animal models for the preclinical assessment of potential therapies for pulmonary fibrosis. Am. J. Resp. Cell Mol. Biol..

[35] Schaefer WH (2004). Evaluation of ubiquinone concentration and mitochondrial function relative to cerivastatin-induced skeletal myopathy in rats. Tox. Appl. Pharmacol..

[36] Seachrist JL, Loi CM, Evans MG, Criswell KA, Rothwell CE (2005). Roles of exercise and pharmacokinetics in cerivastatin-induced skeletal muscle toxicity. Tox. Sci..

[37] Tomaszewski M, Stepien KM, Tomaszewska J, Czuczwar SJ (2011). Statin-induced myopathies. Pharmacol. Rep..

[38] Howard OM (2002). Histidyl-tRNA synthetase and asparaginyl-tRNA synthetase, autoantigens in myositis, activate chemokine receptors on T lymphocytes and immature dendritic cells.. J. Exp. Med..

[39] Tidball JG (2017). Regulation of muscle growth and regeneration by the immune system. Nat. Rev. Immunol..

[40] Deyhle MR, Hyldahl RD (2018). The role of T lymphocytes in skeletal muscle repair from traumatic and contraction-induced injury. Front Physiol..

[41] Zhang J (2014). CD8 T cells are involved in skeletal muscle regeneration through facilitating MCP-1 secretion and Gr1(high) macrophage infiltration. J. Immunol..

[42] Blechynden LM (1997). Myositis induced by naked DNA immunization with the gene for histidyl-tRNA synthetase. Hum. Gen. Ther..

[43] Fernandes-Cerqueira C (2018). Patients with anti-Jo1 antibodies display a characteristic IgG Fc-glycan profile which is further enhanced in anti-Jo1 autoantibodies. Sci. Rep..

[44] Mescam-Mancini L (2015). Anti-Jo-1 antibody-positive patients show a characteristic necrotizing perifascicular myositis. Brain.

[45] Eloranta ML (2007). A possible mechanism for endogenous activation of the type I interferon system in myositis patients with anti-Jo-1 or anti-Ro 52/anti-Ro 60 autoantibodies. Arthritis Rheum..

[46] Guo M, Schimmel P (2013). Essential nontranslational functions of tRNA synthetases. Nat. Chem. Biol..

[47] Bohan A, Peter JB (1975). Polymyositis and dermatomyositis (first of two parts). N. Engl. J. Med..

[48] Griggs RC (1995). Inclusion body myositis and myopathies. Ann. Neurol..

[49] Ashcroft T, Simpson JM, Timbrell V (1988). Simple method of estimating severity of pulmonary fibrosis on a numerical scale. J. Clin. Pathol..

