# Supplementary material for: Serum-circulating His-tRNA synthetase inhibits organ-targeted immune responses
Source: Cell Mol Immunol. 2019 Dec 4;18(6):1463–75. doi: 10.1038/s41423-019-0331-0 (PMC8166958; doi:10.1038/s41423-019-0331-0)
Supplement: Supplementary file 1 — Supplementary Legends [file 41423_2019_331_MOESM1_ESM.docx]

**Supplementary Figure 1: Circulating free HARS levels are specifically altered in anti-Jo-1-positive myositis patients**

(a) Dot blot analysis was performed of serum from healthy individuals, patients with myositis, rheumatoid arthritis (RA) and sarcoidosis (Sarc). The numbers above the blots represent different subjects. Anti-Jo-1 seropositive samples are indicated with a + or – symbol above the subject number. Recombinant HARS is shown as a positive control (rHARS). (b) Free serum HARS levels were determined by ECLIA and are segregated by myositis disease type (Polymyositis (PM), Dermatomyositis (DM), Inclusion body myositis (IBM), Juvenile dermatomyositis (JDM)). (c) Control synthetase levels (left panel GARS, right panel NARS) were determined in anti-Jo-1-positive and -negative myositis patients.

**Supplementary Figure 2: Increase in HARS release during HSkMC differentiation**

(a) HSkMC were seeded at 40x10^3^/cm^2^ on collagen-coated plates and grown in growth medium for 24 hours before changing to differentiation medium. The first day in differentiation medium was regarded as Diff Day 0. Medium was renewed every 2 days and harvested as 0-2, 2-4 and 4-6 medium samples with 3 biological replicates per condition. Cells were fixed on differentiation days 2, 4 or 6 and stained for myotubes and nuclei, followed by analysis of myotube area. (b) Myotube area is shown over the course of differentiation. (c) The fusion index, calculated as number of nuclei within myotube-stained area over total nuclei number, and (d) the nuclear number per imaging field are shown, with a total of 10 or 20 images being used for analysis. (e) HARS proteins in the medium samples were quantified by an in-house HARS ELISA. The results are shown as the mean ± SEM. (f) A significant correlation (p < 0.0001) between myotube area and released HARS levels was observed. (g) Free HARS, MARS and tubulin proteins in whole cell lysates (intracellular) and in concentrated medium (extracellular) were analyzed by western blotting using specific antibodies, which showed an increase of extracellular HARS along with myotube differentiation. Meanwhile, no tubulin or MARS protein was detected in the HSkMC medium. (h) Fusion index and nuclei number are shown (i) for cells in Fig. 2.

**Supplementary Figure 3: Mouse model for anti-Jo-1-positive antisynthetase syndrome reveals differences in muscle function**

(a) Groups of 8 mice were treated using the indicated schedule for immunization and cardiotoxin challenge. CFA = Complete Freund’s Adjuvant, IFA = Incomplete Freund’s Adjuvant. (b) Animals were immunized and challenged with cardiotoxin (CTX) as described above. Six days after cardiotoxin or vehicle administration, animals were briefly suspended by the tail and evaluated by a blinded observer for splaying of the hind limbs (0 = normal, full splay, 1 = impaired, 2 = severely impaired; no splay of the legs from the body).

**Supplementary Figure 4: T cell activity is localized to the WHEP domain of HARS**

Primary human T cells were activated with anti-CD3/anti-CD28 in the presence of HARS or HARS lacking the WHEP domain (ΔWHEP). Treatment with HARS resulted in a significant reduction of IL-2 secretion that was greater than what was seen with ΔWHEP. Error bars represent SEM and statistics by 2-way ANOVA: ns, not significant from vehicle; *, p <0.05, **, p<0.01.

**Supplementary Figure 5: HARS modulates immune checkpoint receptors**

Cell surface expression of PD-1 and CTLA-4 was measured on primary human T cells. HARS treatment resulted in decreased numbers of T cells expressing PD-1 (left panel) and CTLA-4 (right panel) under stimulated conditions (α-CD3/CD28). Error bars represent SEM, and statistics were performed by one-way ANOVA: *, p <0.05, **, p<0.01.

**Supplementary Figure 6: Treatment with HARS improved survival in a severe model of statin-induced myopathy**

(a) Daily dosing of 10-week-old female Sprague-Dawley rats over 14 days with 1 mg/kg cerivastatin reconstituted in 0.5% methylcellulose resulted in very ill rats that showed rapid decline in weight, dehydration and loss of hind limb function. Control rats were dosed daily with 0.5% methylcellulose (no statin). Out of 29 statin-treated rats, 15 were euthanized early due to illness, and 3 were dead between days 10 and 15. Animals dosed starting day 6 with HARS at 0.3, 1 and 3 mg/kg (*, p = 0.0224; Log-rank test) exhibited decreased morbidity and improved survival in a dose-dependent fashion in the less severe model of statin-induced myopathy. (b) Decreased immune cell infiltration was observed in HARS-treated animals and was quantified by the number of total nuclei per 20X field. (c) Decreased expression of genes related to inflammation and immune cell markers was observed in the muscle of HARS-treated animals.
