# Supplementary figures and images for: Serum-circulating His-tRNA synthetase inhibits organ-targeted immune responses
Source: Cell Mol Immunol. 2019 Dec 4;18(6):1463–75. doi: 10.1038/s41423-019-0331-0 (PMC8166958; doi:10.1038/s41423-019-0331-0)

## Supplementary Figure 1

**a**

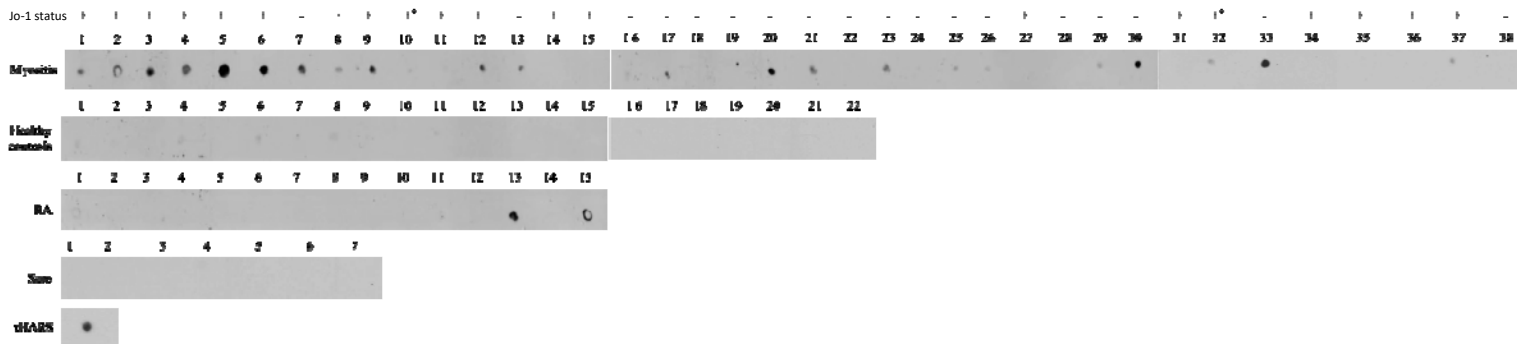**b**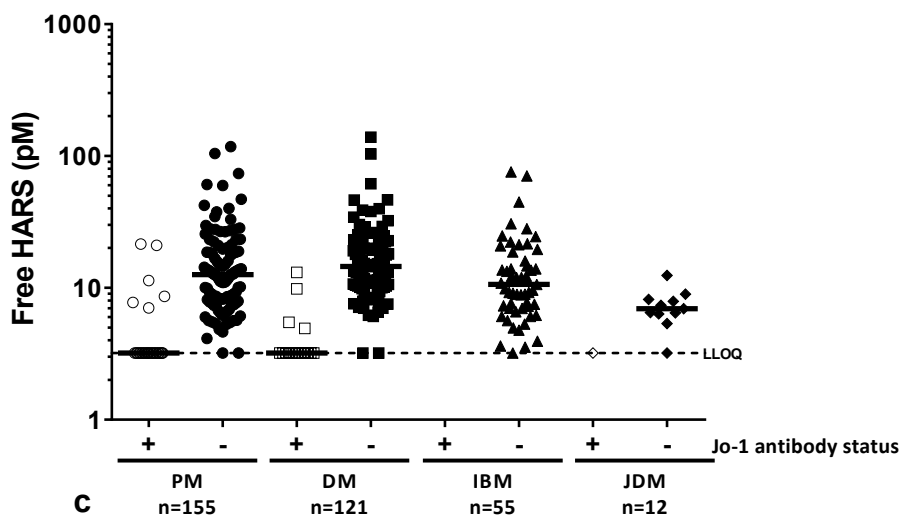

**C**

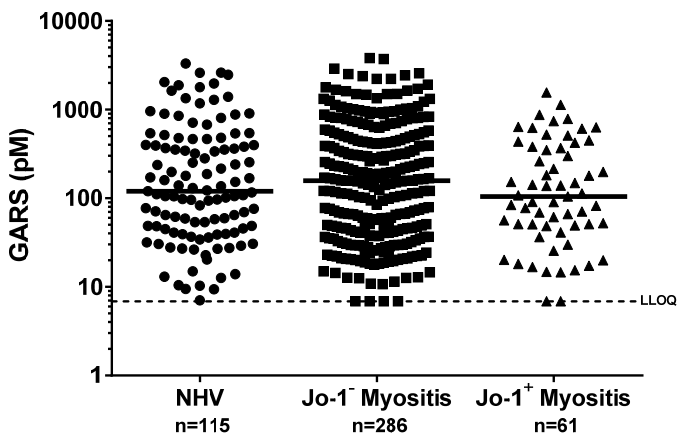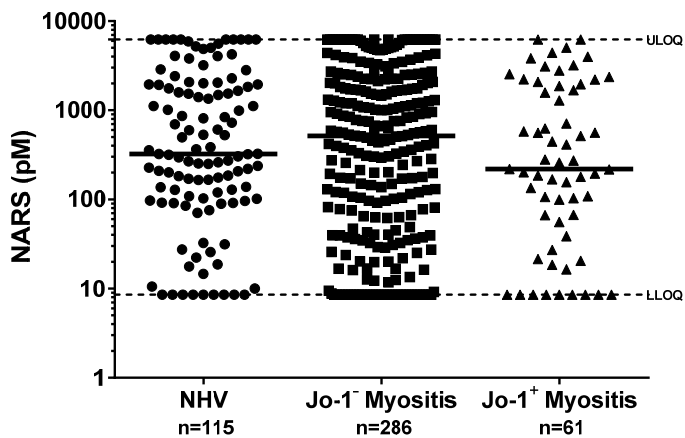

Supplement: Supplementary file 2 — Supplementary Figure 1 [file 41423_2019_331_MOESM2_ESM.pdf]

## Supplementary Figure 2

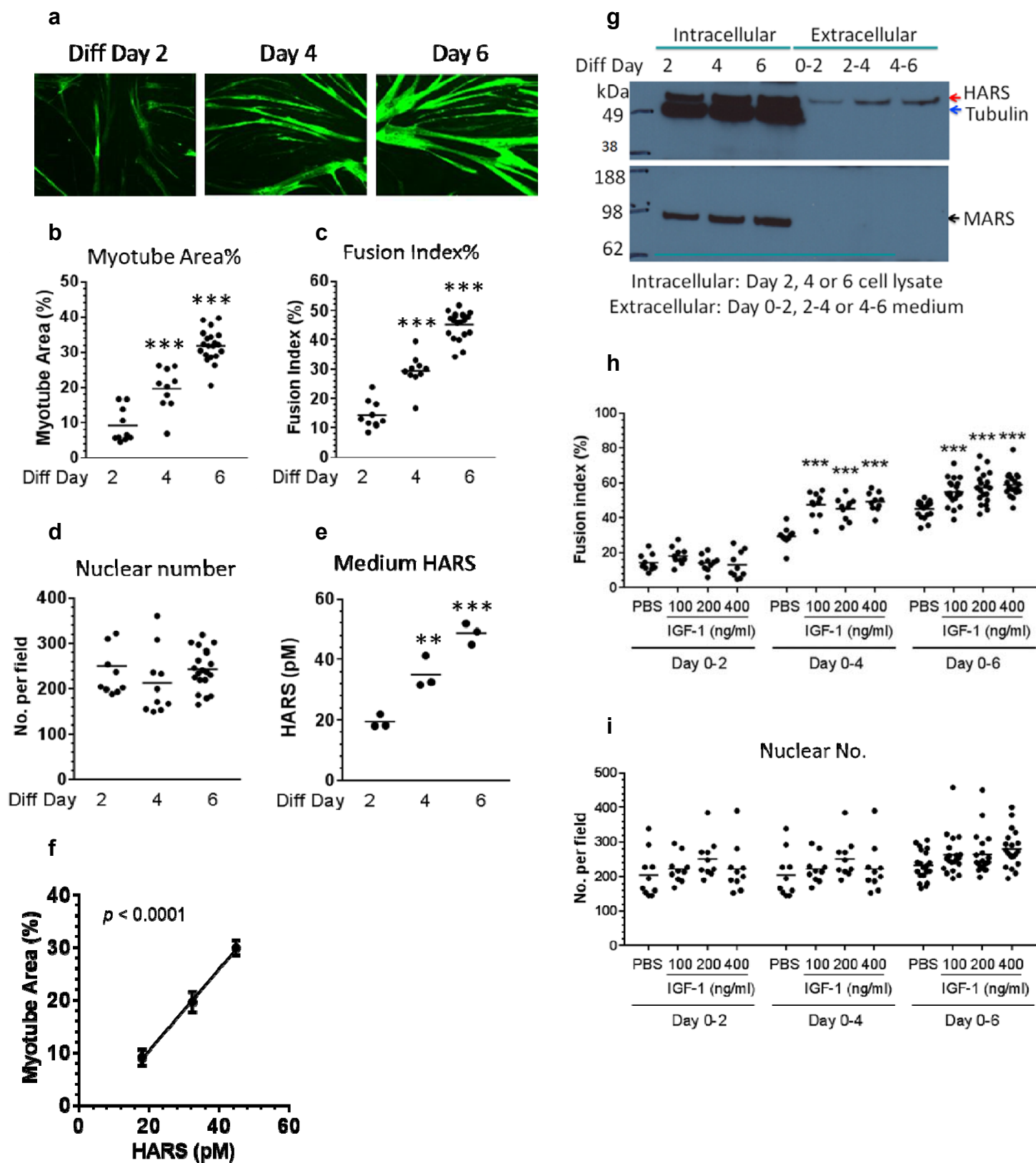

Supplement: Supplementary file 3 — Supplementary Figure 2 [file 41423_2019_331_MOESM3_ESM.pdf]

Supplementary Figure 3

a

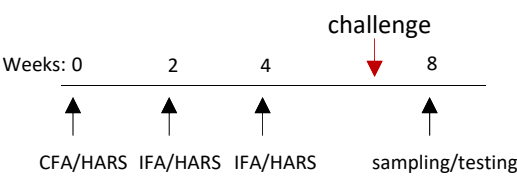

b

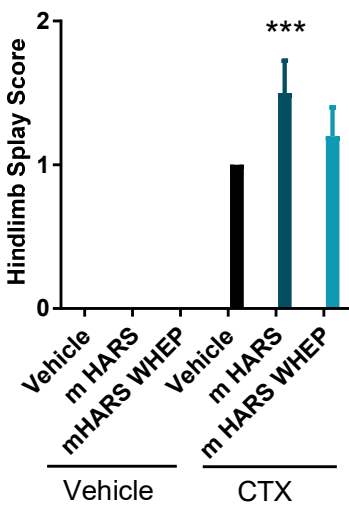

Supplement: Supplementary file 4 — Supplementary Figure 3 [file 41423_2019_331_MOESM4_ESM.pdf]

Supplementary Figure 4

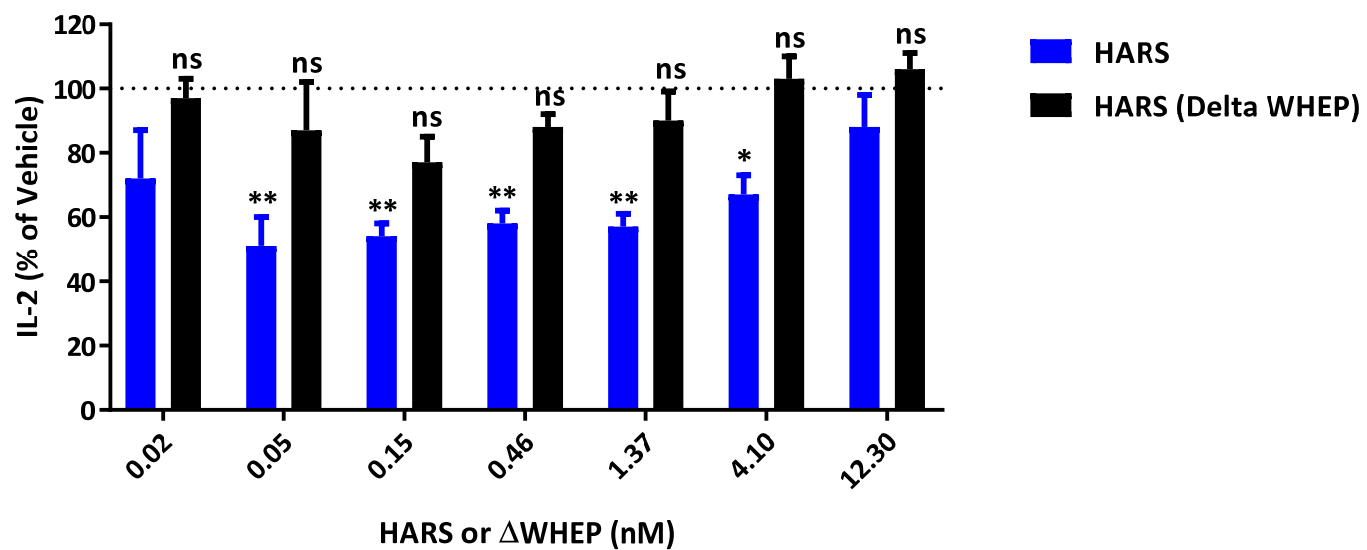

Supplement: Supplementary file 5 — Supplementary Figure 4 [file 41423_2019_331_MOESM5_ESM.pdf]

Supplementary Figure 5

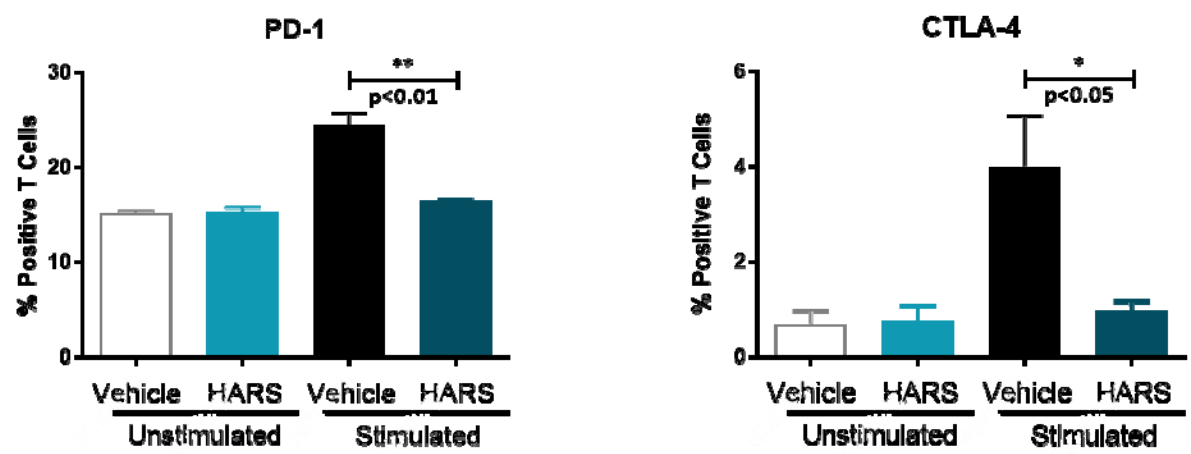

Supplement: Supplementary file 6 — Supplementary Figure 5 [file 41423_2019_331_MOESM6_ESM.pdf]

## Supplementary Figure 6

a

### Mouse Statin Model

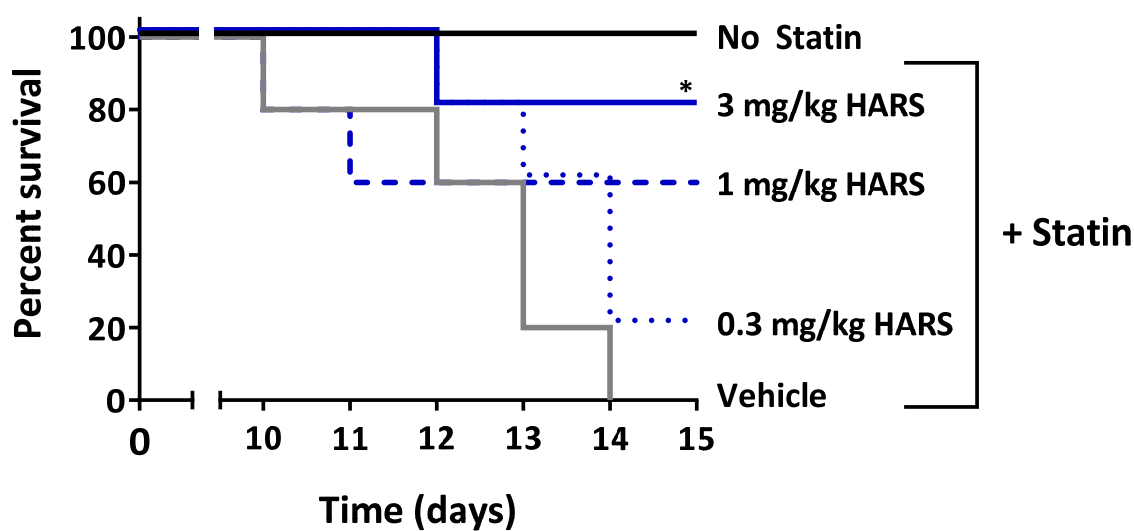

b

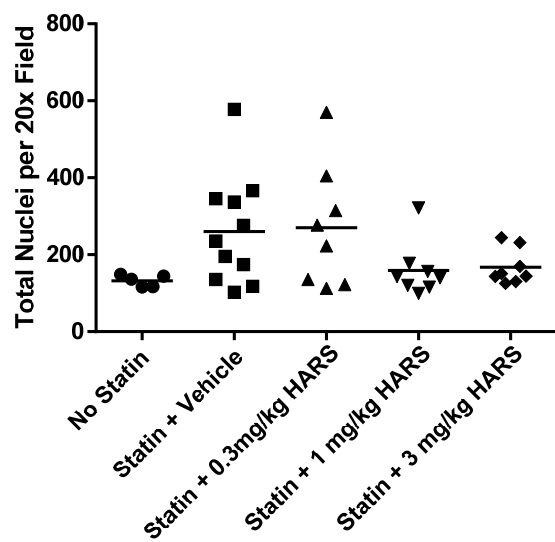

c

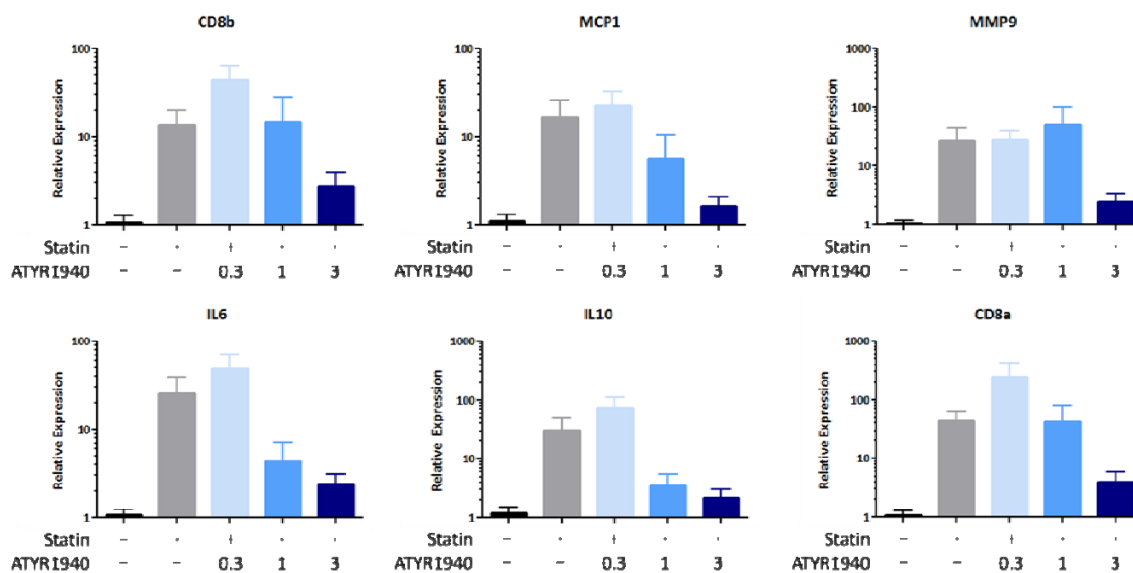

Supplement: Supplementary file 7 — Supplementary Figure 6 [file 41423_2019_331_MOESM7_ESM.pdf]
